# Transcriptional gene silencing in humans

**DOI:** 10.1093/nar/gkw139

**Published:** 2016-04-07

**Authors:** Marc S. Weinberg, Kevin V. Morris

**Affiliations:** 1 Department of Molecular and Experimental Medicine, The Scripps Research Institute, La Jolla, CA 92037, USA; 2 Wits/SAMRC Antiviral Gene Therapy Research Unit, School of Pathology, University of the Witwatersrand, WITS 2050, South Africa; 3 HIV Pathogenesis Research Unit, Department of Molecular Medicine and Haematology, School of Pathology, University of the Witwatersrand, WITS 2050, South Africa; 4 Center for Gene Therapy, City of Hope – BeckmanResearch Institute; Duarte, CA 91010, USA; 5 School of Biotechnology and Biomedical Sciences, University of New South Wales, Kensington, NSW, 2033 Australia

## Abstract

It has been over a decade since the first observation that small non-coding RNAs can functionally modulate epigenetic states in human cells to achieve functional transcriptional gene silencing (TGS). TGS is mechanistically distinct from the RNA interference (RNAi) gene-silencing pathway. TGS can result in long-term stable epigenetic modifications to gene expression that can be passed on to daughter cells during cell division, whereas RNAi does not. Early studies of TGS have been largely overlooked, overshadowed by subsequent discoveries of small RNA-directed post-TGS and RNAi. A reappraisal of early work has been brought about by recent findings in human cells where endogenous long non-coding RNAs function to regulate the epigenome. There are distinct and common overlaps between the proteins involved in small and long non-coding RNA transcriptional regulatory mechanisms, suggesting that the early studies using small non-coding RNAs to modulate transcription were making use of a previously unrecognized endogenous mechanism of RNA-directed gene regulation. Here we review how non-coding RNA plays a role in regulation of transcription and epigenetic gene silencing in human cells by revisiting these earlier studies and the mechanistic insights gained to date. We also provide a list of mammalian genes that have been shown to be transcriptionally regulated by non-coding RNAs. Lastly, we explore how TGS may serve as the basis for development of future therapeutic agents.

## INTRODUCTION

### The history of RNA-directed transcriptional gene silencing (TGS)

**Figure 1.**
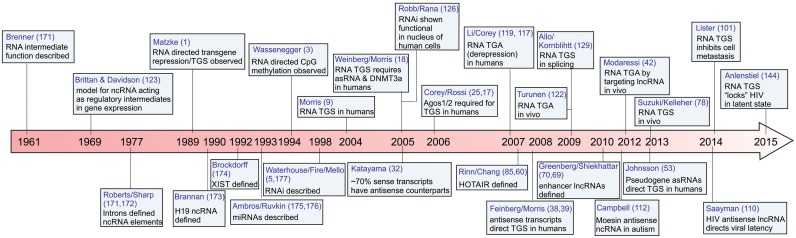
Regulatory non-coding RNA timeline. A timeline of some important observations in RNA biology are shown leading up to our collective understanding of non-coding RNA-directed transcriptional gene silencing (TGS). (5,71,170–177).

Almost three decades ago, Marjorie Matzke *et al*. observed that over-expression of a transgene led to DNA hypermethylation and transcriptional silencing in doubly transformed tobacco plants (1) (Figure 1). Mechanistically, this type of silencing in plants was found to be the result of small non-coding RNAs directing epigenetic changes, specifically DNA methylation, to those loci containing sequences homologous to the small RNA. The phenomenon was termed small RNA-directed transcriptional gene silencing (TGS). TGS was later shown in *Arabidopsis* to require the action of RNA-dependent DNA methylation (2,3) and members of the Argonaute protein family (4). A few years later RNA interference (RNAi), mediated by double-stranded RNAs, was discovered as a powerful post-TGS (PTGS) system against messenger RNAs (mRNAs) in plants (5), and a few months later in *Caenorhabditis elegans* (6).

### Transcriptional gene silencing in humans

The study of small non-coding RNA-directed TGS has been carried out in various model organisms such as plants (*Arabidopsis thaliana*), yeast (*Saccharomyces pombe*), flies (*Drosophila melanogaster*) and worms (*C. elegans*) (reviewed extensively in (7,8)). A decade ago, the first report of RNA-directed TGS in human cells was observed when exogenous siRNAs were used to silence a transgenic elongation factor 1 α promoter driving a Green Fluorescent Protein (GFP) reporter gene (9) (Figure 1). Importantly, the observed silencing was clearly at the transcriptional level, as indicated by nuclear run-on analysis. Moreover, silencing was also epigenetic: inhibition was abrogated by 5′ Aza-cytadine (5′ AzaC) and Trichostatin A (TSA), compounds involved in inhibiting DNA methylation and histone deacetylalion, respectively (9). This early observation was soon followed by other studies (10,11), all of which confirmed that small non-coding RNAs could functionally control gene transcription and epigenetic states in human cells. But the underlying mechanism of action remained unknown.

### Mechanisms of small non-coding RNA-directed TGS

**Figure 2.**
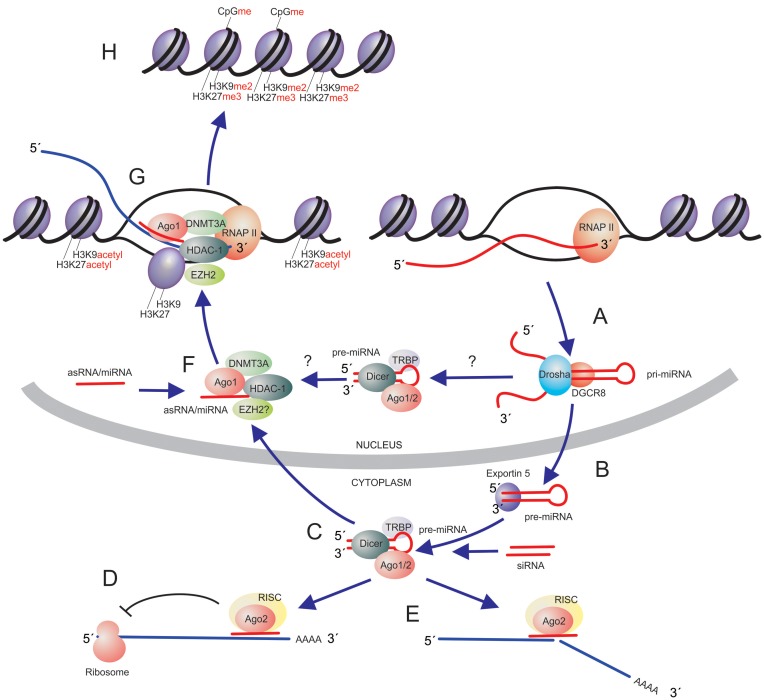
Small non-coding RNA pathways in human cells. Small non-coding RNAs can be generated as priRNAs where they are (**A**) processed by Drosha and DGCR8 into miRNAs which are (**B**) exported from the nucleus and (**C**) loaded into RISC where they can affect mRNA expression by (**D**) binding and blocking mRNA translation or (**E**) cutting the target mRNA. Some miRNAs may also be retained in the nucleus (**F**) where they can interact with epigenetic remodeling proteins and (**G**) recruit the complexes to target loci in the genome resulting in (**H**) localized chromatin compaction and epigenetic silencing.

TGS is mechanistically distinct from the abundantly studied PTGS pathway of RNAi. One notable difference is that TGS results in long-term stable epigenetic modifications to gene expression that can be passed on to daughter cells during cellular division (reviewed in (12)). Early observations postulated that siRNA-directed TGS functioned through an epigenetic nuclear mechanisms distinct from RNAi-mediated PTGS in the cytoplasm (13). For instance 5′ AzaC and TSA were functional in reverting the siRNA targeted TGS, indicating epigenetic modes of gene regulation were at play in siRNA-directed TGS, and not via a PTGS-based mechanism (9). Indeed, recent studies have observed that two different siRNAs, one targeted to the promoter and one targeted to exon 1 of the coding transcript, can functionally repress the targeted gene in a TGS or PTGS based manner (14). A lot has been gleaned over the last decade regarding the mechanism of action for RNA-directed TGS in human cells. Studies carried out to determine the underlying mechanism of siRNA-directed TGS revealed that RNA-mediated TGS is operative through RNA-directed methylation of histone 3 lysine's 9 and 27 (H3K9 and H3K27, respectively) and DNA methylation at the targeted promoter (9,11,15–21) (Figure 2). These promoter-directed siRNAs interact with a low level expressed (∼1–2%) promoter associated RNA, which is essentially the 5′ UTR of the protein coding gene (16,22) (Figure 2). It is worth noting that most genes and gene promoters appear to be transcribed to some extent (23,24) and experimental observations suggest that non-coding RNAs interact with target loci via Watson–Crick-based RNA:RNA hybridization (16,22) and not by double-stranded DNA invasion. Temporal studies have determined that exogenously introduced siRNAs targeted to a promoter region interact first with Argonautes 1 and 2 (AGO1 and AGO2) (17,25,26). SiRNA and AGO interactions is found within the first 24 h, at the siRNA targeted promoter and is followed shortly thereafter with the recruitment of the H3K9me2 and H3K27me3 silent state epigenetic marks (17), and later by the recruitment of DNA methyltransferase and DNA methylation at 72–96 h for some genes (14). It should be noted, however, that the role of DNA methylation in TGS in human cells is not as clearly understood as in plants; DNA methylation at the targeted promoter is not always observed in human TGS applications (Table 1). These effects may be explained by the duration of RNA targeting to the promoter, the presence of robust siRNA targeting (e.g. delivery to the nucleus), the presence and abundance of promoter-occupied RNAs and/or the dynamic interplay of proteins interacting with the promoter. Despite differences in the various experimental observations, a key consistent feature has been the observations that promoter-directed small RNAs can modulate gene transcription and that some level of epigenetic based silencing is ongoing in the observed silenced genes.

**Table 1.** Mammalian genes transcriptionally regulated by non-coding RNAs

Gene(s)	Gene symbol	Effector RNA	Cell line	Therapeutic relevance	References
Eukaryotic translation elongation factor 1 α	EEF1A1	siRNA	HEK293T		(9,16,18)
HIV-1/SIV	5′ LTR	siRNA, sasRNA	Jurkat, Tzmb, T-cells, *in vivo* (mouse)	Regulation of HIV-1	(18–21,26,79,99,100,104,140–144)
nitric-oxide synthase	NOS	siRNA	HAEC	Cardiac disease	(145)
E-cadherin	CDH1	siRNA	HCT116; MCF7	Cancer, tumor suppressor	(10)
BCL-2 (oncogene)	BCL-2	sasRNA	HeLa, 293	Cancer, oncogene	(146)
Fibronectin	FN1	siRNA	HeLa		(129,147)
Huntingtin gene	HTT	siRNA	Glioblastoma	Monogenetic diseases	(148)
Non-sense codon-containing immunoglobulin minigenes	(Ig)-mu and Ig-gamma	siRNA	HeLa	Immunologic diseases	(149)
INK4B/Cyclin-dependent kinase inhibitor 2B/p15 + ARF + INK4A/ Cyclin-dependen kinase inhibitor 2A isoform 3/p16	CDKN2B+ CDKN2A	siRNA	HEK293T	Cancer, tumor suppressor	(150)
INK4A/Cyclin-dependen kinase inhibitor 2A isoform 3/p16	CDKN2A	siRNA	HEK293T	Cancer, tumor suppressor	(151)
Plasminogen activator, urokinase	PLAU	siRNA	PC3 and *in vivo*	Cancer	(152)
Chemokine receptor 5	CCR5	siRNA	HEK293T	HIV-1 co-receptor	(16,17)
Breast cancer-associated gene 1	BRCA1	siRNA	T47D	Cancer, oncogene	(153)
Progesterone receptor	PGR	siRNA	T47D	Cancer	(11,25,119,153,154)
Huntingtin	HD	siRNA	T47D	Monogenetic diseases	(25)
Androgen receptor	AR	siRNA	T47D	Cancer, spinal bulbar muscular atrophy	(25,155)
v-myc avian myelocytomatosis viral oncogene homolog	c-MYC	siRNA/sasRNA	PC3, HCT113, 293, Hela, MCF7	Cancer, oncogene	(22,97,156,157)
Papillomavirus-16 oncogenes	HPV-16	siRNA	HeLa	HPV	(158)
v-akt murine thymoma viral oncogene homolog 1	AKT-1	siRNA	293HEK	Cancer, oncogene	(156)
Kirsten rat sarcoma viral oncogene	KRAS	siRNA	293HEK	Cancer, oncogene	(156)
Dual specificity phosphatase 6	DUSP6	siRNA	CFPAC	Cancer, tumor suppressor	(156)
Myostatin	MSTN	siRNA	C2C12 mouse myoblasts	muscle hypertrophy	(159)
Runt-related transcription factor 3	RUNX3	siRNA	stomach carcinoma cell line SGC7901	osteoarthritis	(160)
Small nuclear 7sk (RNAi functional in the nucleus of human cells)	7SK	siRNA	Hela, 293HEK cells	7SK/P-TEFb control of HIV-1	(126)
met proto-oncogene (hepatocyte growth factor receptor)	c-Met	siRNA/asRNA	SKHep1C3 cells	Cancer, oncogene	(161)
Periostin	POSTN	siRNA/sasRNA	PC3	Cancer and metastasis	(101)
Heparanase (endo-h-D-glucuronidase)	HPA	siRNA	PC3, EJ and SGC-7901 cells	Cancer, angiogenesis	(162)
phosphoglycerate kinase 1 promoter driving GFP	pgk-1	siRNA	293, HeLa		(98)
Interleukin 2	IL2	shRNA	Jurkat	Immunologic	(163)
Ubiquitin C	UBC	siRNA; shRNA	HEK293GT		(14)
Transforming growth factor-β receptor II	TGFβII	shRNA	rat SBC10	Cancer, angiogenesis	(102)
Vascular endothelial growth factor	VEGF-A	shRNA	mouse C166 and *in vivo*	Cancer, angiogenesis	(107,122)
Ras association domain family 1	RASSF1A	shRNA	HeLa	Cancer, oncogene	(15,17)
Tubulin folding cofactor E-like	TBCEL/ LRRC35	miRNA	HCT116	Kenny-Caffey syndrome (KCS)	(150)
Ras p21 protein activator 2	RASA2	miRNA	HCT116	Cancer, tumor suppressor	(150)
Rhophilin, Rho GTPase binding protein 2	RHPN2	miRNA	HCT116		(150)
Wolf-Hirschhorn syndrome candidate 1	WHSC1	miRNA	HCT116	Wolf-Hirschhorn syndrome	(150)
Homeobox D4	HOXD4	miRNA	MCF7; MDA-MB-231		(164)
HIV-1	LTR	miRNA	Jurkat, T-cells	HIV-1 infection	(28)
HIV-1	TAR	miRNA	Tzmb, Jurkat, T-cells	HIV-1 infection	(29,165)
OCT4 and Nanog (pluripotent factor)	OCT4 and Nanog	lncRNA (antisense, pseudogene)	MCF7	Cancer, pluripotency	(54)
PTENpg1 asRNA alpha	PTEN	lncRNA (Trans-antisense, pseudogene)	293, Hela, Jurkat	Cancer, tumor suppressor	(53)
P21 tumor suppressor	P21	lncRNA (antisense)	MCF7	Cancer, tumor suppressor	(39)
P15 tumor suppressor	P15	lncRNA (antisense)	HL-60, KG-1, Kasumi-1, DG-75, Raji and Ramos	Cancer, tumor suppressor	(38)
alpha-globin gene	HBA2	lncRNA	Embryonic stem cells	Alpha thalassemia	(40)
DM1 insulator	DM1/SIX5	lncRNA	Primary fibroblasts		(166)
Herpes	LAT	lncRNA	*in vivo* (mice)	HPV	(167)
P53, lncRNA-p21	hnRNP-K	lncRNA	MEF, *in vivo* (mice)	Cancer, oncogene	(57)
lncRNA HOTAIR	HOX	lncRNA	MDA-MB-231, SK-BR-3, MCF-10A, MCF-7, HCC1954, T47D and MDA-MB-453 cell lines. Human tissue samples	Cancer	(86)
HIV-1	HIV-1	lncRNA (antisense)	Jurkat, T-cells, Tzmb, 293HEK	HIV-1 infection	(49–52)
v-myc avian myelocytomatosis viral oncogene neuroblastoma derived homolog	MYCN MYCNOS	lncRNA (*Cis*-antisense)	Lan6	Cancer, oncogene, neuroblastoma	(43)
neuroblastoma associated transcript 1	NBAT-1	lncRNA	Neuroblastoma primary tumors	Cancer, neuroblastoma	(48)
brain-derived neurotrophic factor	BDNF	lncRNA (antisense)	Human brain, mouse *in vivo*	Huntington disease, Alzheimer disease and Parkinson disease	(41,42)
short-chain dehydrogenase/reductase family member 4	DHRS4	lncRNA (antisense)	HepG2 and HL7702 cell		(44)
potassium voltage-gated channel, KQT-like subfamily, member 1	Kcnq1ot1	lncRNA (antisense)	Human placenta-derived JEG-3 cells	Romano-Ward syndrome, Jervell and Lange-Nielsen syndrome and familial atrial fibrillation	(168)
moesin at 5p14.1 in Autism	Moesin	lncRNA (antisense)	Human brain (postmortem cerebral cortex)	Autism spectrum	(113)
C-terminal binding protein 1	CTBP1	lncRNA (antisense)	LNCaP, VCaP, DU145	Prostate cancer	(169)

Reports from 2004 to present using exogenously administered small interfering RNAs (siRNAs), small hairpin RNAs, small antisense RNAs (sasRNA) and endogenously expressed microRNAs (miRNAs) or long non-coding RNAs (lncRNAs) effector transcripts to modulate gene transcription in mammalian cells are shown. Those genes targeted and their therapeutic relevant disease is also shown.

### The endogenous pathway of TGS in human cells; rise of long non-coding RNAs

While small RNAs were observed early on to regulate gene transcription in human cells by the targeting of epigenetic silencing complexes to those loci containing complementarity to the small RNAs (Figure 1), the endogenous mechanism(s) driving this form of gene regulation in the context of human cells remained largely unknown. MicroRNAs (miRNAs) have been shown to be endogenous drivers of TGS with some genes in human cells (27–30)(Table 1). In 2005, through the efforts of the FANTOM and ENCODE consortia, it started to become apparent that a large fraction of the human genome was generating long non-coding RNAs (lncRNAs) and that many of these transcripts were antisense to protein-coding counterparts (31,32). Several of these sense/antisense or bidirectionally-transcribed genes are evolutionarily conserved, suggesting some functional cues for retention of these elements (33,34). Indeed, studies with imprinted genes and X-inactivation found that *cis* acting long non-coding RNAs (lncRNAs) were actively involved in epigenetic regulation of these, dosage-dependent, regulated loci (35).

In 2008, a number of important studies confirmed the role of lncRNAs as endogenous drivers of TGS in human cells, in particular those, which were antisense to their protein-coding counterparts (reviewed in (36,37)). Antisense lncRNAs were shown to regulate the p15 (38) and p21 (39) tumor suppressor genes (Table 1). The over-expression of these antisense lncRNAs resulted in TGS of their protein-coding counterpart while their repression resulted in the de-repression/transcriptional activation (38,39). Support for the role of antisense lncRNAs as active endogenous regulators of gene expression was evident in an earlier understated study, which indicated that antisense transcripts might also be involved in CpG methylation in thalassemia (40). Antisense lncRNAs are now known to affect TGS for genes such as BDNF (41,42), MYCN (43), DHRS4 (44), KCNQ1 (45–47), NBAT (48) and HIV-1 (49–52) (Table 1). Interestingly, non-coding transcripts derived from pseudogenes of Phosphatase and tensin homolog (PTEN) (53) and OCT4 (54), which contain significant homology to their protein-coding counterparts, have also been observed to be involved in directing TGS and subsequent PTEN and OCT4 suppression (Table 1). Also, the PTEN antisense pseudogene-directed TGS of PTEN is one of the first *bona fide* examples of a trans-functional lncRNA (53). LncRNAs direct TGS and CpG methylation (55). *Cis* regulation and antisense lncRNAs have also been observed to epigenetically regulate ribosomal genes (56) as well as p21 (57) and MYCN (43), genes involved in cell regulation and cancer. The plethora of lncRNA functions are extensive and the range includes: protein modifiers (58,59), scaffolds for tethering proteins (43,60,61), miRNAs (62–64), splicing modifiers (65), cellular body transformation (66–69), enhancer function and gene activation (70–73), and epigenetic modifiers (53,74,75), (reviewed in (76)). Collectively, the observations to date suggest that we are only now just beginning to realize the complexity and pervasiveness of lncRNA functional regulation in epigenetic and transcriptional states.

### Mechanisms of small and long non-coding RNA-directed TGS

**Figure 3.**
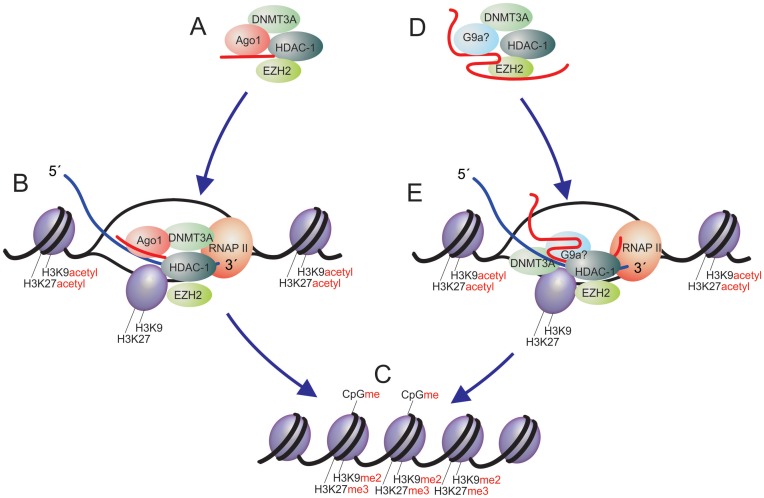
Antisense RNA-directed TGS. Small antisense non-coding RNAs can be (**A**) introduced into the nucleus and (**B**) interact with and recruit epigenetic silencing complexes consisting of DNMT3a, Ago1, EZH2 and HDAC1 to homology containing targeted loci by interactions with low copy promoter-associated transcripts resulting in (**C**) epigenetic silencing consisting of histone and DNA methylation and ultimately chromatin compaction of the targeted locus. (**D**) Long antisense non-coding RNAs have also been observed to interact with similar epigenetic silencing complexes (53,54) and (**E**) localize with these complexes at targeted loci resulting in (C) epigenetic silencing of the lncRNA targeted locus.

To date there are ∼55 reports of small RNA-directed TGS and ∼10 of antisense lncRNA-directed TGS (Table 1). Mechanistically, much of what we know about how small non-coding RNAs, such as siRNAs, miRNAs and small antisense RNAs (sasRNAs)-directed TGS, have been determined from cell culture studies. The promoter-targeted small RNAs interact with various proteins to guide TGS, beginning in the first 24 h, with direct interactions with AGO1 and AGO2 (17,18,25) followed shortly thereafter by interactions at the targeted promoter with DNMT3a (14,18,77,78), HDAC1 (14,20) and resulting ultimately in histone 3 lysine 9 di-methylation and histone 3 lysine 27 tri-methylation (H3K9me2 and H3K27me3, respectively)(14,16–18,20,79,80) (Figure 3). SiRNA-directed TGS has also been observed to occur in the absence of DNA methylation, suggesting that alternative routes may be present for RNA-mediated transcriptional and epigenetic silencing (10). Small RNA-directed TGS appears to require a template or target transcript at the corresponding targeted promoter (16,22), similar to the method by which plants utilize RNA Polymerase V transcribed and processed siRNAs to regulate DNA methylation and TGS (reviewed in (81)). Notably, in plants there is a requirement for RNA-dependent RNA polymerase (RdRP) activity to amplify RNA polymerase V transcript-directed TGS (81), whereas humans lack such a polymerase, which opens up a methodology for specific RNA-directed epigenetic modes of regulation. Curiously, this is exactly what lncRNAs appear to be doing in human cells via *cis* and trans-specific targeting of epigenetic complexes to particular loci (Figure 3 and Table 1), similar to what is also observed in *Saccharomyces cerevisiae*, which also lacks RdRP activity (82,83).

Early studies carried out with *S. cerevisiae* indicated that antisense non-coding RNAs function endogenously to direct epigenetic gene silencing in place of RdRP-mediated mechanisms (82,83). The parallels between *S. cerevisiae* and previous observations of small antisense RNA-directed TGS in human cells (18) have emerged, suggesting that antisense transcripts also function to direct TGS (Figure 3). Most notable are the observations that particular antisense lncRNAs, first observed in tumor suppressor genes, p15 (38) and p21(39), function to epigenetically modulate their protein-coding counterparts (Figure 1, Table 1). One interesting, and surprisingly overlooked, early study found antisense transcription was involved in DNA methylation in Thalassemia (40), and even early work linked antisense transcripts and DNA methylation in regulating HIV (51) and MYC (84,85).

Mechanistically, far less is known about how antisense lncRNAs direct epigenetic silencing in human cells. Studies carried out with the lncRNA, HOTAIR, indicate that bimodal chromatin modifying complexes can be localized to the HOX locus via the action of this lncRNA (86). A common theme is also evident with Kcnq1ot1 (45) and the p53 regulatory lincRNA p21, which indicates that the entire p53 expressed pathway is controlled by the action of this lncRNA at the p53 locus (57). Indeed, many lncRNAs have been observed to be associated with chromatin (87), but mechanistic insights into the process of lncRNA-directed gene regulation remain less clear. Interesting insights into the mechanism of action of lncRNA-directed TGS came from a recent study looking at the PTEN pseudogene. It had been reported previously that the PTEN pseudogene functions as a miRNA ‘sponge’ (64), similar to the CEBPA lncRNA that acts to sponge DNMT1 away from the *CEBPA* promoter (88). Studies to interrogate the PTEN pseudogene in greater detailed determined that this pseudogene also expressed an antisense lncRNA in *trans* which functions to direct TGS to the PTEN promoter and control PTEN expression epigenetically (53). Mechanistically, the PTEN pseudogene expressed antisense lncRNA modulated PTEN transcription by recruiting DNMT3a and EZH2 to the PTEN promoter. The parallels between the functions of the PTEN pseudogene and previous observations with small antisense ncRNA-directed TGS are notable, as both involved the action of DNMT3a (Figure 3). It is noteworthy that DNMT3a is the only known *de novo* DNA methyltransferase in human cells (89) and has been observed previously to be the only DNA methylatransferase to bind non-coding RNAs including small ncRNAs, both antisense and double stranded RNAs (18,77,78,90), and lncRNAs (53,91). There is an interesting connection between DNMT3a and epigenetic silencing, which including studies indicating DNTM3a co-immunoprecipitates with HDAC1 (92,93) and EZH2 (94), as well as early predictions that DNA methylation is an active participant in X-inactivation (95), one of the first *bona fide* lncRNA regulatory pathways described. Collectively, a paradigm is emerging in human cells, which proposes that non-coding RNAs, both small and long forms (Figure 3), function through the action of DNMT3a to modulate chromatin and epigenetic states of gene expression. While there are several other mechanisms of action described for lncRNAs in human cells, the interactions with DNMT3a and targeting of transcriptional and epigenetic states is of particular interest, as this mode of gene regulation has the potential to be long-lasting, heritable and may be of significant relevance to the development of targeted therapeutics (reviewed in (96)).

### Therapeutic applications of RNA-directed epigenetic regulation of gene expression

The utility of small RNA-induced TGS as a therapeutic has been largely ignored, mainly due to the pervasiveness of using RNAi targeted approaches to degrade mRNAs. The main concern with RNAi and post-transcriptional mechanisms of gene silencing (Figure 2) is the duration of their therapeutic effect. The effector siRNAs required to drive RNAi must be administered continuously to repress a therapeutic target gene. This is not the case with RNA-induced TGS, where stable, long-term, silencing can be achieved following a relatively short duration of promoter targeting with the siRNAs (19,20,97–100) or small antisense RNA (14,101). This is because the mode of action for the observed gene silencing is transcriptional and driven ultimately by epigenetic silencing (79,102) and not ‘slicing’ of the genes messenger RNA as is the case with RNAi. One universal hurdle that both RNAi and RNA-induced TGS face with is the targeted delivery of the effector RNAs to those cells requiring treatment. One approach is to utilize synthetic antisense oligonucleotides targeted to promoters of interest. This approach has worked with regards to blocking transcription (103) but was not found to induce robust epigenetic silencing, unless the particular oligonucleotides were RNA based (104). However, it may be that better interrogation of each non-coding RNA targeted promoter is required to delineate the best promoter-associated transcripts to target and that many of the earlier studies may have neglected this notion. Indeed, establishing TGS in the absence of a target promoter RNA has not been reported and attempts by some groups, including ours, have proven fruitless. Another approach might be to deliver the effector RNAs using receptor targeted aptamers, which has shown promise for targeting HIV infected cells (105,106). While delivery remains an important concern, the notion that one needs to only target a particular gene for 2–4 days to instill stable epigenetic silencing is promising with regards to minimizing the need for sustained delivery. Recent studies suggest that small RNA-directed TGS is feasible and that stable epigenetic marks can be imposed at small RNA target loci *in vivo* (99,107).

**Figure 4.**
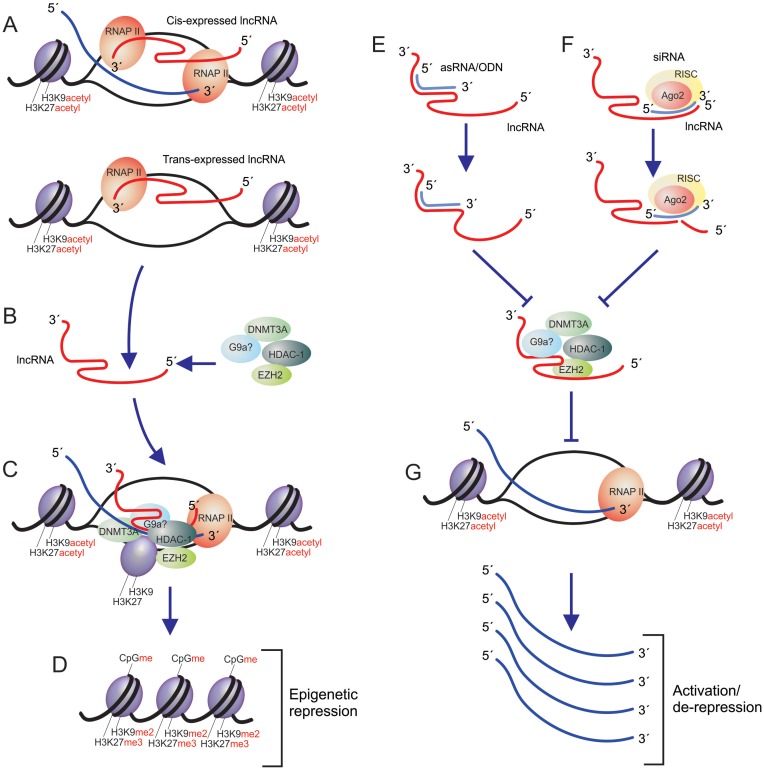
LncRNA pathways of transcriptional silencing and de-repression. LncRNAs can be expressed in (**A**) *Cis* or *trans* and can (**B**) interact with those proteins involved in epigenetic silencing. The lncRNAs act to (**C**) target and tether the epigenetic silencing complexes to homology containing loci resulting in (**D**) chromatin compaction and transcrtiptinal gene silencing of the targeted locus. These endogenous regulatory lncRNAs can be targeted with (**E**) antisense oligonucleotides or (**F**) siRNAs, which results in the loss of the lncRNA and activation/de-repression of those loci actively under lncRNA regulation.

Another area of therapeutic utility can be found in the plethora of lncRNAs that are appearing to be involved in various diseases. Emerging evidence suggest that non-coding RNAs play a wide role (108) in various disease states in humans. Genome-wide observations of diseased states, such as heart failure (109), indicate significant differential and discordant expression between protein-coding and non-coding antisense and pseudogenes is prevalent (110). To date the list of those lncRNAs involved in human diseases is expanding at an unprecedented rate. LncRNAs have been observed in disease ranging from Cancer (57,86,105,106), to HIV (111,112), to autism (113), to pluripotency and differentiation (114–116). It is worth underscoring that many of the disease relevant lncRNAs have been observed to be antisense to particular protein coding genes. A significant obstacle to using RNAi and other post-transcriptional effectors for targeting antisense lncRNAs is the fact that double stranded siRNAs have an ability to target both sense and antisense transcripts (117). The use of RNA-directed TGS avoids this issue by targeting the lncRNA promoter with single stranded antisense transcripts (52). The targeting of endogenous effector antisense lncRNAs can result in the de-repression and subsequent transcriptional activation of the lncRNA targeted locus (Figure 4). Using this mode of action, it becomes feasible to activate gene expression to affect those protein-coding genes under sustained lncRNA-directed TGS (Figure 4). This has proven an effective approach to inducing genes both *in vitro* (39,52,118–121) and *in vivo* (42,107,122), but presupposes that there are known antisense lncRNAs regulating the therapeutic target gene. Collectively, the advantages to using RNA-directed TGS as a therapeutic are many and include: (i) strand specific targeting of a gene, (ii) stable long-term epigenetic based silencing can be established to particular genes of therapeutic interest and (iii) antisense RNA-based approaches work as well, if not better than double stranded RNAs, as the endogenous pathway of RNA-directed TGS appears to contain significant overlap with small antisense RNAs and antisense lncRNAs (Figure 3).

### CONCLUSION

It has been roughly 10 years since the first observation that promoter-directed RNAs can affect gene transcription (Figure 1 and Table 1). This seminal observation in 2004 (9) was indicative of a role for RNA in regulating gene expression, a notion proposed ∼5 decades ago but largely overlooked (123,124). Possible reasons for the poor early adoption of RNA-directed TGS (Table 1) are varied but may include (i) the unfortunate retraction of a similar paper published in Nature (125), and/or (ii) the overwhelmingly positive response to PTGS and the rejection of any RNAi-related phenomena occurring in the nucleus, despite the fact that RNAi was shown to be functional in the human nucleus in 2005 (126) and confirmed in many subsequent studies (25–27,127–131).

The notion that RNA may function as the master gene regulator in the cell was something proposed by Britten and Davidson in 1969 (123), which at the time was largely neglected by the broad scientific community. With the advent of high-throughput technologies and the findings from ENCODE, that most of the human genome is transcribed and likely plays a functional role (132–139), it is becoming apparent that Britten and Davidson's theory should be reappraised. Certainly, lncRNAs are abundantly active in the nucleus, and many of them are active modulators of transcriptional and epigenetic modes of gene expression (reviewed in (37,76) and appear to share many of the mechanistic characteristics observed in small RNA-directed TGS (Figure 3). Collectively, the mounting observations that antisense non-coding RNAs, both small and long RNAs, directed to gene promoters can affect transcription by the recruitment of silent state epigenetic complexes suggests that a pervasive and underappreciated role for non-coding RNAs is part of the basic fabric of life. Knowledge of this molecular pathway may prove incredibly insightful with regards to the development of disease, including epigenetic silencing of gene expression and the development of new-targeted therapeutics aimed at specifically affecting gene expression. The next decade could prove an exciting time for our understanding of non-coding RNAs in the transcriptional gene expression and their application as novel therapeutics.
